# Next Generation Sequencing of miRNAs – Strategies, Resources and Methods 

**DOI:** 10.3390/genes1010070

**Published:** 2010-06-03

**Authors:** Susanne Motameny, Stefanie Wolters, Peter Nürnberg, Björn Schumacher

**Affiliations:** 1 Cologne Center for Genomics (CCG), University of Cologne, 50931 Cologne, Germany; 2 Cologne Excellence Cluster for Cellular Stress Responses in Aging Associated Diseases (CECAD), University of Cologne, 50674 Cologne, Germany

**Keywords:** miRNA, next generation sequencing

## Abstract

miRNAs constitute a family of small RNA species that have been demonstrated to play a central role in regulating gene expression in many organisms. With the advent of next generation sequencing, new opportunities have arisen to identify and quantify miRNAs and elucidate their function. The unprecedented sequencing depth reached by next generation sequencing technologies makes it possible to get a comprehensive miRNA landscape but also poses new challenges for data analysis. We provide an overview of strategies used for miRNA sequencing, public miRNA resources, and useful methods and tools that are available for data analysis.

## 1. Introduction 

microRNAs (miRNAs) are small RNA molecules of 17 to 24 bp that play an important role in the regulation of gene expression by modulating translation and stability of mRNA. Next generation sequencing technologies have enabled sequencing of the complete set of miRNAs present in an RNA sample. The data obtained from such sequencing experiments can be used to characterize miRNA expression and function by expression profiling, identification of sequence isoforms, prediction of novel miRNAs, and prediction of potential mRNA target molecules. In this review, we address the basic data processing steps that are involved in miRNA sequencing experiments and give an overview of available methods to perform the respective type of data analysis. Furthermore, we list public miRNA resources that provide useful information for the analysis of miRNA sequencing experiments. We restrict the description of the technical details of miRNA sequencing to the Illumina Genome Analyzer platform, but the data analysis strategies are similar for other available next generation sequencing technologies and will differ only in technical details of the initial data processing.

## 2. The Biology of miRNAs

miRNAs as well as short interfering RNAs (siRNAs) mediate RNA interference (RNAi) by binding to the 3’-UTR of target mRNAs either resulting in repressed translation or mRNA decay [1,2,3]. This endogenous regulation system is mediated by incomplete complementarity between the miRNA and the mRNA target sequence. miRNAs modulate cell function in all tissues and control development and differentiation. Furthermore they are part of a large variety of signaling cascades and can also alter the cell cycle (for review, see [2]). 

**Figure 1:**
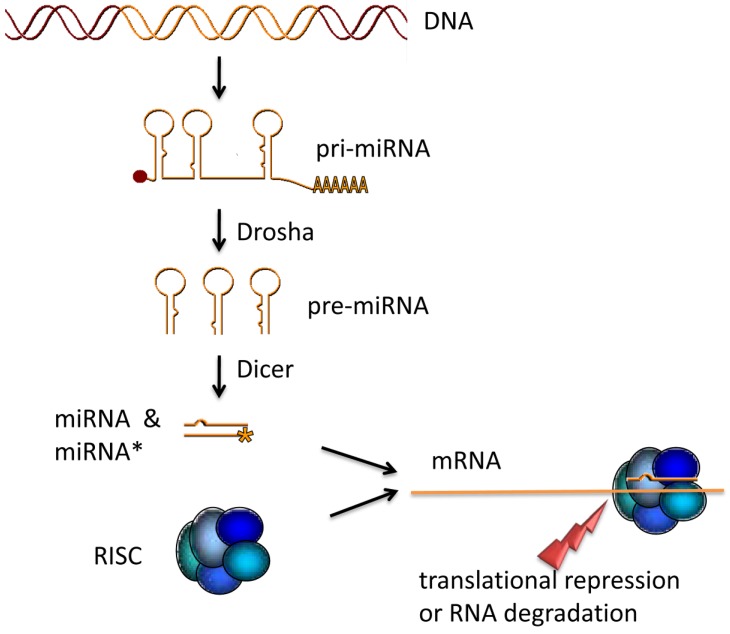
miRNA maturation. The primary miRNA transcript is processed by the endoribonuclease Drosha. Thus generated precursor miRNAs are transported into the cytoplasm and cut into 24 bp fragments by Dicer. After the double stranded miRNA::miRNA* fragment is loaded into the RNA Induced Silencing Complex (RISC) the miRNA* is degraded. The RISC containing the mature miRNA binds a target mRNA to inhibit translation either by repression of translation or by mRNA degradation.

About 40% of all known miRNAs are encoded in intronic sequences [3]. The rest is either coded in intergenic regions or organized in clusters. The primary transcript of a miRNA gene (pri-miRNA) can be over 1kb long and has a 5’-Cap and a Poly-A-tail [1] (Figure 1). The pri-miRNA possesses one or more miRNA-containing stemloops. A microprocessor complex comprising a class II endoribonuclease III Drosha cuts the pri-miRNA into a shorter (60-70bp) precursor miRNA (pre-miRNA) [4]. After the precursor is actively transported into the cytoplasm another endoribonuclease Dicer cuts off the loop region and produces a ~22 bp long double stranded RNA, which contains the mature miRNA and its complementary strand called miRNA* [5]. Although in most cases the miRNA* is degraded when the miRNA is incorporated in an effector complex, it has been suggested that the miRNA* can act as a miRNA as well [6]. Generally, miRNA* is found less frequently in the sequencing data than the mature miRNA [7]. The effector complex contains members of the Argonaute protein family and several other factors, which together build an isoform of the RNA-Induced Silencing Complex (RISC) (for review, see [2]). By binding of this ribonucleoprotein complex mediated by the miRNA to target sequences in the 3’-UTR of an mRNA, translation is repressed. It has been shown that a high complementarity between the 2^nd^ and 8^th^ base at the 5’-end of the miRNA and the binding site of the mRNA is crucial for the function of the miRNA [8]. This region of the miRNA is known as seed region and is conserved across species and within miRNA families. The conservation of the seed region is the basis for many target prediction algorithms.

## 3. Next Generation Sequencing of miRNAs using the Illumina Genome Analyzer

**Figure 2:**
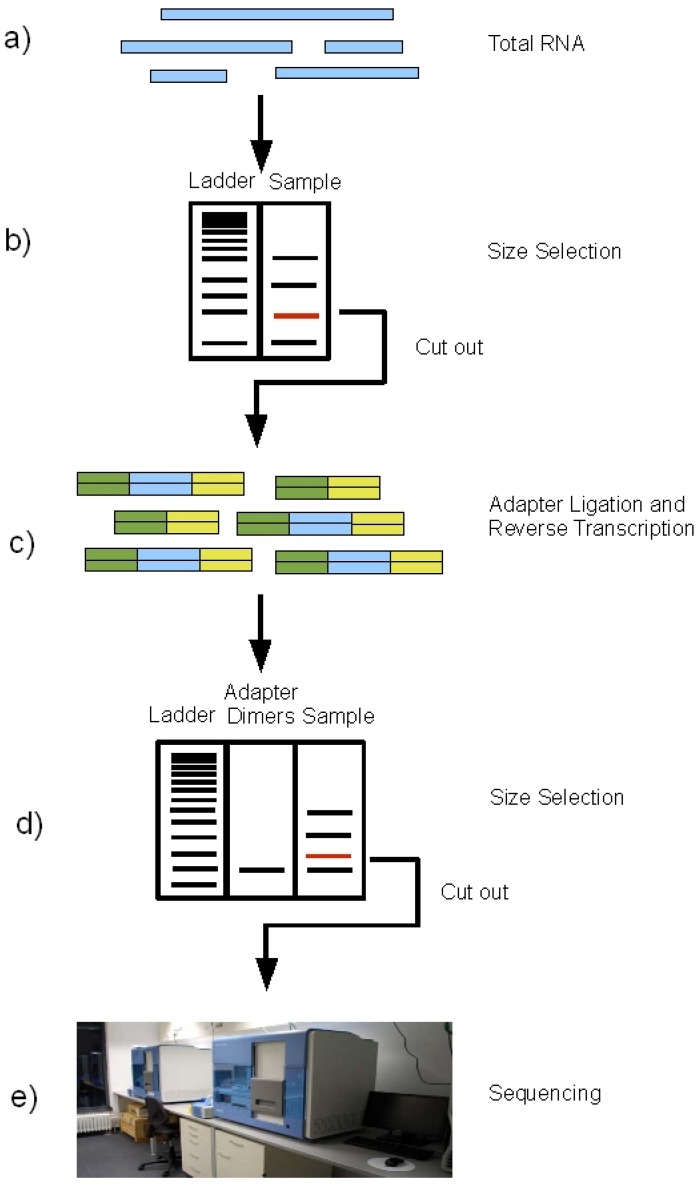
Schematic of the miRNA sequencing procedure on the Illumina Genome Analyzer. **a)** Extraction of total RNA, **b)** Size selection, **c)** Adapter ligation and reverse transcription, **d)** Size selection, **e)** Sequencing.

The “wet-lab” part of miRNA sequencing comprises several steps. First, the total RNA is extracted from the sample (Figure 2). For the preparation of the RNA either a spin column based kit like miRVana (Ambion) or miRNeasy (Qiagen), which allow also preparation of the small RNA fraction can be used, or TRIzol preparation following ethanol precipitation can be performed. Standard column based RNA preparation kits common for mRNA preparation should be avoided because this normally leads to the loss of smaller RNA molecules. Furthermore, it is useful to not only isolate the small RNAs because in this case the rRNA fraction cannot be used for an RNA integrity analysis. It is essential to confirm the quality of the RNA before sequencing to make sure that biologically relevant oligonucleotides are sequenced and degradation products do not influence the results. To extract the miRNA fragments from the total RNA, a size selection is performed: the total RNA is run on an agarose gel and the band corresponding to the size of miRNAs is cut out for further processing. This procedure excludes all bigger fragments, including all mRNAs and also rRNAs from the samples. In the next step, the sequencing adapters are ligated to the size-selected RNA molecules, followed by reverse transcription to cDNA. The thus obtained cDNA library is run on an agarose gel again and the band containing the molecules corresponding to the miRNA fragments with ligated adapters is cut out for subsequent sequencing. To avoid contamination with adapter dimers in this step, an additional control sample, containing adapter dimers only and a size ladder allowing the isolation of cDNA species larger than adapter dimers is run in parallel to the cDNA library. 

## 4. Basic Data Processing Steps

The output of a next generation miRNA sequencing experiment will typically contain millions of short reads. Before specific research questions can be addressed, several basic data preprocessing steps have to be performed to extract the relevant information from these raw data.

### 4.1. Quality Filtering and Alignment to a Reference Genome 

The reads from a next generation sequencing experiment are commonly provided as textfiles in FASTQ format. Such files contain four lines per read: the first line contains the “@” symbol followed by a (unique) read identifier; the second line contains the read's nucleotide sequence; the third line again contains the read identifier, this time preceded by the “+” symbol, or just a plain “+”, and the fourth line contains quality scores that specify the probability that the nucleotide call is wrong for each nucleotide in the read sequence [9]. Missing nucleotides in the sequence are usually denoted by the “N” character. Based on the quality scores, an initial filtering step can be performed to exclude reads of low quality as well as reads that contain too many missing nucleotides. However, most erroneous reads will be filtered out by one of the following data processing steps anyway, so that in many cases such initial quality filtering can be skipped.

A minimum requirement for each sequence is that the read should originate from the genome of the sequenced organism. This is particularly relevant when for instance the experimental organism is co-cultivated with a feeder organism. Therefore, as an initial step, all produced reads are aligned to the reference genome of the sequenced organism and all reads whose first part (15 to 17 bp to allow for sequence isoforms that show single-nucleotide 3' extensions [10]) perfectly matches the reference are kept as potential miRNA reads. The remaining reads are discarded from further analysis. For this alignment step it is convenient to use a short read aligner like maq [11], soap [12,13], eland (part of the Illumina Pipeline Software), or bwa [14] that readily identify perfect matches at much higher speed than the more versatile aligners like blast [15] or megablast [16]. Note that some of the short read aligners consider reads that match the reference at several positions to be repeats and do not report such alignments by default. This has to be prevented by adequate parameter settings in order to avoid the loss of relevant reads. Table 1 summarizes commonly used software and database tools for the various analysis steps described in this review.

To directly identify known miRNAs, sequences can be aligned with those of annotated miRNAs. An up to date list, which is commonly used for miRNA experiments is provided by miRBase [17] for many different organisms. 

### 4.2. Removal of 3' Sequencing Adapter

The sequence reads start at the first base after the 5' sequencing adapter (*i.e.*, the first base of the original cDNA molecule) and in Illumina sequencing typically end after 36 bp. As mature miRNAs are normally only up to 24 bp in length, the reads will contain part of the 3' adapter sequence that has to be removed. Tools for this purpose include the trimLRPatterns function contained in the Bioconductor Biostrings package [18] for the R programming language [19] and a BioPerl script (available at http://www.bioperl.org/wiki/Removing_sequencing_adapters). The novoalign alignment software (http://novocraft.com) has an option to automatically trim adapter sequences from the reads. It is also convenient to align all reads against the adapter sequence using a flexible aligner like lastz or megablast [16] and use the alignment information to trim the adapter sequence off the reads. This strategy is preferable particularly if there are reads in the data that were sequenced through the complete 3' adapter and carry additional “tails” of unknown or highly error-prone sequence after the adapter sequence (typically occurring when longer reads of 50 bp and more are produced). In this case, the available tools for adapter trimming frequently miss the adapter sequence whereas aligners will reliably detect it. 

**Table 1:** miRNA Online Resources and Tools.

1. Alignment Tools		
lastz	http://www.bx.psu.edu/miller_lab/	Flexible alignment tool
megablast [16]	http://blast.ncbi.nlm.nih.gov/Blast.cgi?CMD=Web&PAGE_TYPE=BlastDocs&DOC_TYPE=Download	Flexible alignment tool
maq [11]	http://maq.sourceforge.net/maq-man.shtml	Short read aligner
soap [12,13]	http://soap.genomics.org.cn/	Short read aligner
bwa [14]	http://bio-bwa.sourceforge.net/	Short read aligner
**2. miRNA Analysis Tools**		
miRExpress [20]	http://mirexpress.mbc.nctu.edu.tw/index.php	miRNA expression profiling
miRDeep [21]	http://www.mdc-berlin.eu/en/research/research_teams/systems_biology_of_ gene_regulatory_elements/projects/miRDeep/index.html	miRNA prediction tool from sequencing datasets
miRTools	http://centre.bioinformatics.zj.cn/mirtools/	miRNA profiling and discovery
TargetScan [22]	http://www.targetscan.org/	Online software for microRNA target identification
PicTar [23]	http://www.mdc-berlin.eu/en/research/research_teams/systems_biology_of_gene_regulatory_elements/projects/pictar/index.html	Algorithm for miRNA target prediction
miRanda [24]	http://www.microrna.org	Algorithm for miRNA target prediction
DIANA microT [25]	http://diana.cslab.ece.ntua.gr/	Online software for microRNA target identification
RNAHybrid [26]	http://bibiserv.techfak.uni-bielefeld.de/rnahybrid	Algorithm for miRNA target prediction
miTarget [27]	http://cbit.snu.ac.kr/~miTarget	Algorithm for miRNA target prediction
microRNA.org [28]	http://www.microrna.org/microrna/home.do	Prediction of targets and expression
mirWIP [29]	http://146.189.76.171/query.php	Algorithm for miRNA target prediction
MicroCosm Targets [17]	http://www.ebi.ac.uk/enright-srv/microcosm/htdocs/targets/v5/	miRNA target prediction (formerly miRBase Targets)
miRecords [30]	http://mirecords.biolead.org/	Database for validated and predicted miRNA targets
**3. Databases**		
mirBase [17]	http://www.mirbase.org/	Database for miRNA research
deepBase [31]	http://deepbase.sysu.edu.cn/	platform for next generation miRNA data analysis
TarBase [32]	http://diana.cslab.ece.ntua.gr/tarbase/	Database for known interactions between miRNA and target mRNAs
miR2Disease [33]	http://www.mir2disease.org/	Resource of miRNA deregulation in various human diseases
PMRD [34]	http://bioinformatics.cau.edu.cn/PMRD/	Plant miRNA database
**4. General Tools**		
R [19]	http://cran.r-project.org/	Free software environment for statistical computing and graphics
Perl	http://www.cpan.org/	Programming language
Vienna Package [35]	http://www.tbi.univie.ac.at/RNA/	RNA secondary structure prediction

### 4.3. Filtering of Other RNA Species

In addition to reads of mature miRNAs, the sequence data will most probably also contain reads from various other RNA species, including other non coding small RNA species and RNA degradation products. It is reasonable to filter out reads that align against such sequences prior to further downstream analysis in order to simplify the interpretation of the results. The sequences of such small RNA species like rRNA, small cytoplasmic RNA, small nuclear RNA, small nucleolar RNA, tRNA and protein coding regions can be found on the University of Santa Cruz (UCSC) Genome Browser.

## 5. Expression Profiling

A topic that is frequently addressed by miRNA sequencing is the quantitative comparison of miRNA expression in two or more samples. For this purpose, expression levels are computed based on the read counts in each sequenced sample. For each unique sequence among the reads, the number of times it occurs among all the reads of the sample is computed and normalized against the total number of reads that were produced for the sample. A common way to do this is to compute the *rpm* (Reads Per Million) value of each sequence *s* occurring in the sample according to the following formula.

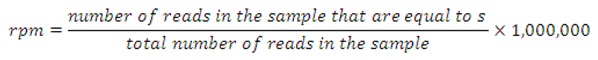



In theory, these normalized read counts should be a direct measure for the amount of fragments of the respective sequence in the sample and therefore its expression level. However, it has been shown that the fragment composition of the sample is significantly altered depending on the methods used for RNA extraction and library preparation [36]. The absolute normalized read counts are therefore not representatives of expression levels. As in microarray analysis, the analysis is limited to relative comparisons of normalized read counts between samples to detect expression changes. 

Prior to performing expression analysis, sequencing errors have to be removed. On the Illumina Genome Analyzer platform, single base substitution errors are the main concern. Assuming that the errors occur at random positions of the sequence and the substituted nucleotide is also selected randomly, sequences containing errors are expected to have low read counts. Indeed it has been shown that when looking at the distribution of read counts of all reads there is a big proportion found less than 1-10 times [37]. Filtering out all sequences with read counts less than a low threshold in each sample is therefore a common strategy to eliminate sequencing errors. Usually, the threshold that is used for this filtering step is chosen arbitrarily. In [37], the authors suggest a statistical method to determine the threshold automatically. They iteratively compare the cumulative distribution functions of read frequencies between replicate samples for different thresholds until the similarity between the distributions is satisfyingly high. To determine differentially expressed sequences, the established methods from the analysis of microarray data are then used on the filtered sequences. These include the computation of fold-changes if the experiment contains only two samples, the two-sample t-test if the experiment contains two groups of samples, or ANOVA if three or more groups of samples are involved. In order to approximate the normality assumption that underlies most of the statistical methods mentioned above, the logarithmized normalized read counts should be used for these analyses. A freely available tool that performs such kinds of expression analyses is miRExpress [20]. MirTools and deepBase [31] offer web-based platforms for next generation miRNA data analysis that also include expression analysis.

The sequencing results can be verified performing quantitative real-time PCR. Since miRNAs are only about ~22 bp long they cannot be detected in a normal RT-qPCR, thus special approaches have been developed for this purpose. TaqMan® MicroRNA Assays from AppliedBiosystems are using miRNA-specific stemloop primers for reverse transcription of the miRNAs followed by qPCR using primers and TaqMan® MGB probe specific for the respective small RNA. In this case a reverse transcription reaction for each miRNA to be detected in a sample needs to be performed. However, if several miRNAs are to be detected in one sample another technique, which elongates small RNAs during the reverse transcription, is recommendable. One example for such a product is miScript from Qiagen. During reverse transcription RNAs are polyadenylated and transcribed into cDNA using oligo-dT primers and random primers. The oligo-dT primers have a universal tag sequence on the 5'-end, which allows amplification of the small RNAs during qPCR. miRNA specific forward primers (which are in most cases identical to the respective miRNA) and miScript universal reverse primers targeting the universal tag are used in a SYBR Green real-time PCR to quantify the respective miRNA in the samples. Furthermore, small RNAs that are not differentially regulated in all samples should be taken for normalization. Often the U6 RNA or the 6S rRNA are recommended for this purpose but unaltered expression of these molecules in the sequencing results should be verified and furthermore the C_T_ of the control RNA in the different samples after qPCR should be compared.

## 6. Identification of Isoforms

Within the data obtained from a next generation miRNA sequencing experiment, many sequences will typically occur that are identical for all but a few nucleotides. Such sequences might represent different isoforms of the same miRNA. Different types of miRNA isoforms have been described before, including isoforms that may arise from variability of Dicer and Drosha cleavage positions within the pre-miRNA and isoforms showing single-nucleotide 3' extensions leading to mismatches with the reference genome [10]. The origin and function of such isoforms is poorly understood up to now but their presence suggests as yet unknown cellular mechanisms of miRNA processing. When analyzing miRNA sequencing experiments, isoforms can complicate the analysis process as well as the interpretation of the results. In expression analysis, for example, it is not immediately clear which of the different isoforms should be used in the expression comparison, especially if the expression changes of different isoforms show contrary behavior. Currently, it is common practice to consider only the isoform with the highest read count which seems to be a reasonable strategy for now but the handling of isoforms deserves greater attention in the future. 

## 7. Prediction of novel miRNAs

**Figure 3:**
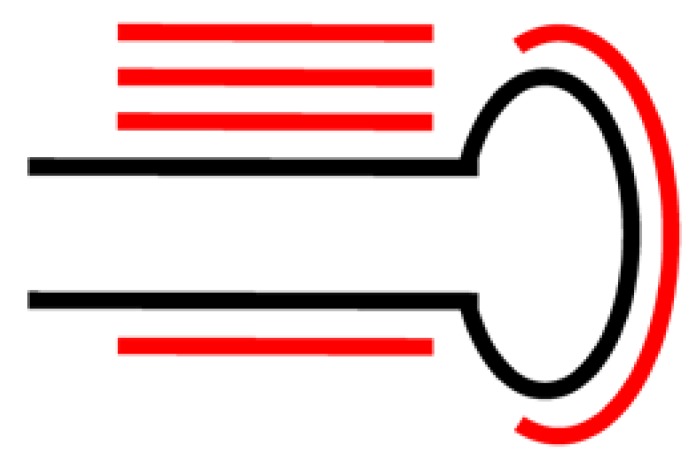
Alignment pattern expected from miRNA processing (Adapted from [38]).

A number of tools are available that aim at predicting novel miRNAs from next generation sequencing data. The predictions made by these tools are generally based on the current biological knowledge of the miRNA processing mechanism in living cells. One of the most commonly used prediction tools is mirDeep [21] that specifically looks for the pattern the miRNA processing mechanism leaves in the sequencing data. The most important pattern in this context that miRDeep considers are clusters of reads that align along the reference genome in a fashion that is compatible with the *mature miRNA sequence – loop sequence – star sequence* structure (Figure 1) of the miRNA precursor molecule as shown in Figure 3. If such a pattern is found, miRDeep cuts out the potential miRNA precursor sequence from the reference genome and utilizes an RNA folding algorithm from the Vienna Package [35] to judge whether the sequence can be folded into a hairpin structure.

Furthermore, the prediction software searches for potential cleavage sites of Drosha and Dicer. The phylogenetical conservation and filtering of other known small non-coding RNA species can be optionally used to improve the predictions. To confirm the presence of the predicted miRNA in the sample quantitative real-time PCR can be performed using the protocols described in *5. Expression Profiling*. 

## 8. Prediction of miRNA Targets

The prediction of the mRNAs that are targeted by known or novel miRNAs is based on comparisons between the sequences of mature miRNAs and sequences of mRNA candidate targets. miRNAs bind to the 3' UTR of their mRNA target which triggers the repression of translation or the degradation of the mRNA depending on the complementarity between the target and the miRNA [3]. High complementarity between the binding region of the target and the miRNA normally leads to the degradation of the target, whereas mismatches in the binding region lead to translational repression.

The tools that are available for miRNA target prediction seek putative binding sites in the 3' UTRs of the candidate mRNAs taking into account the seed region of the miRNA and the conservation between species. Many of the algorithms, like microRNA.org [28] or TargetScan [22] also allow searching for putative binding miRNAs for given mRNA sequences. Additional algorithms for miRNA target predictions include PicTar [23], RNAHybrid [26], miTarget [27] or DIANAmicroT [25]. Since all these programs are slightly different each of them will predict a different group of target mRNAs and a different probability that an mRNA might be a target of a respective miRNA. These deviations in target predictions emphasize the importance of experimental target verification.

For target prediction all known transcripts of the sequenced organism can be used as the set of candidate mRNAs. However, as a given miRNA might regulate different sets of target mRNAs depending on biological context, *i.e.*, tissue specificity or in dependence on signaling events, it has proven useful to compare miRNA expression to mRNA expression analysis. As the target mRNA is typically degraded upon miRNA binding, an inverse correlation between miRNA and target mRNA abundance is expected. The limitation of this approach is that miRNAs often rather modulate the abundance of mRNAs or even inhibit translation as opposed to promoting mRNA degradation. Consequently, mRNA expression data might be insufficient or even misleading when used for the identification of miRNA targets. 

When predicting targets it is important to consider that one mRNA molecule might contain several binding sites for one or more miRNAs and, furthermore, a single miRNA might regulate several targets. The complexity of mRNA-miRNA interactions thus poses challenges to target predictions from sequencing data. 

In addition to the available software for miRNA target prediction, there are also databases where both predicted and experimentally confirmed miRNA targets are collected. A useful tool to analyze known interactions between miRNA and target mRNAs is provided by TarBase [32]. This database allows searches based on miRNA, gene and organism. As output it shows whether the specific mRNA is cleaved or repressed by the binding of the miRNA under study. Furthermore, it offers information about the validity of the miRNA-mRNA interaction by classifying the interaction as TRUE when reduced protein levels have been shown, FALSE when the target gene expression remained unaffected, WEAK when only a low downregulation could be observed or pSILAC/MicroArray when the interaction has been concluded from high-throughput experiments only. In addition to the number of miRNA-mRNA interaction sites present in the respective 3’-UTR it provides further information about the miRNA and mRNA expression and outcomes of deregulation.

The MicroCosm Targets database hosted by EBI contains predicted miRNA targets for the miRBase [17] miRNA sequences. It currently contains predicted miRNA targets for 22 species. Initial target predictions are obtained using the miRanda software [24] followed by inspection of folding properties (computed by the Vienna Package [35]) and conservation across species. 

**Figure 4:**
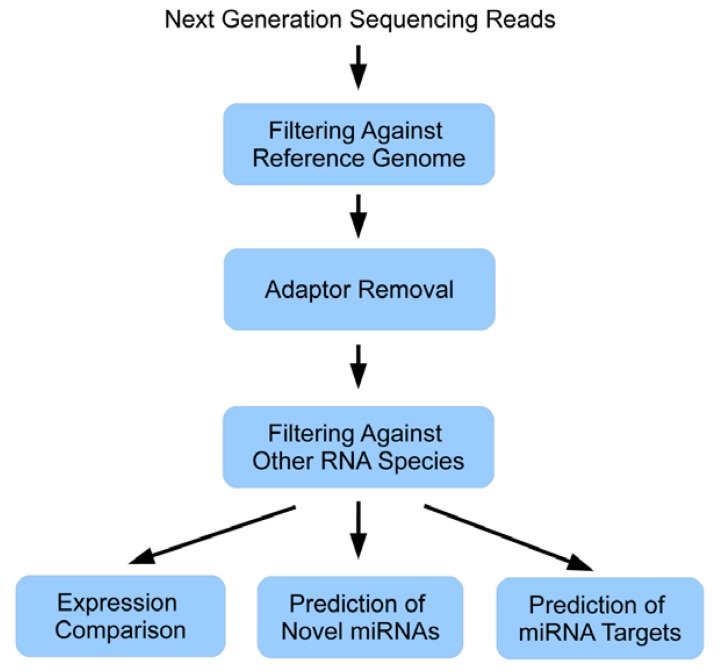
General data analysis workflow of a miRNA next generation sequencing experiment.

Another database containing both predicted and validated targets for currently 9 species is miRecords [30]. It contains miRNA target predictions obtained from 11 different target prediction tools. A database especially dedicated to miRNAs in plants is PMRD [34]. It not only contains miRNA sequences and targets but also expression profiles from several published studies. Figure 4 summarizes the general data analysis workflow of a miRNA next generation sequencing experiment as described in this paper.

## 9. Potential and Limitations of Next Generation Sequencing Compared to Microarray and pPCR Analysis

Prior to the availability of deep sequencing technologies, microarrays were used for expression analyses of miRNAs. A study using artificial miRNA pools with known RNA fragment concentrations showed a high correlation between expression values derived from microarray and deep sequencing [39]. While microarrays were found to better reproduce the fragment concentrations in the artificial pools, deep sequencing technologies have the advantage that novel sequences can be detected while microarrays can only interrogate fragments of known sequence. The advantage of next generation sequencing platforms over microarray hybridization techniques is their enormous sensitivity and dynamic range gained by the high sequencing depth. Even fragments that occur only a few times in the library will be visible in the data and the read counts do not show the saturation effects common to microarray derived expression values. 

For the detection of differential expression between different samples, everything points to differences in sensitivity and reproducibility of data [40]. The comparison of deep sequencing-based expression analysis with different microarray platforms showed not only a higher sensitivity in the detection of transcripts with low expression but also higher expression changes are found by a sequencing approach. Furthermore, every cDNA fragment produces exactly one read so cross hybridization effects do not occur and even fragments that differ by a single nucleotide can be distinguished. Thus, highly similar sequences like miRNA family members can be distinguished by sequencing in contrast to microarray or qPCR experiments. Generally the discrimination of mature and unprocessed forms of miRNAs is a problem miRNA qPCR assays and microarrays are dealing with. By contrast miRNA family members, precursors as well as miRNA modifications can be easily identified by deep sequencing. Also the short length of miRNAs displays a problem for microarray and qPCR design. Often the entire miRNA sequence must be used as a probe for hybridization. Because of this there are variations in the melting temperature between the different probes. Next generation sequencing is independent of predesigned probes, thus making it very suitable for the discovery of new miRNAs. Nevertheless, deep sequencing is a relatively novel approach and the associated computational analysis tools are still in their infancy and need to be improved to standardize normalization, mapping and thresholding.

## 10. Conclusions and Perspectives

miRNAs have been shown to regulate a host of biological processes in plants, nematodes, insects and mammals (for review, see [2]). The advent of high throughput sequencing methodologies has provided unprecedented opportunities to generate comprehensive sequencing data for the identification and quantification of known and novel miRNAs. These technological leaps forward pose new challenges for the biological interpretation of large sequencing data sets. Further investigation of the molecular mechanisms through which miRNAs regulate gene expression will provide important parameters for target identification and thereby the prediction of biological outcomes of miRNA expression.
